# Temporal Activity and Distribution of the Invasive Mosquitoes *Aedes albopictus* and *Aedes japonicus* in the Zagreb Area, Croatia

**DOI:** 10.3390/tropicalmed9110263

**Published:** 2024-11-04

**Authors:** Ana Klobučar, Mihaela Kavran, Sunčica Petrinić, Marcela Curman Posavec

**Affiliations:** 1 Medical Entomology Laboratory, Vector and Pest Control Unit, Department of Epidemiology, Andrija Štampar Teaching Institute of Public Health, Mirogojska c. 16, 10000 Zagreb, Croatia; marcela.curman@stampar.hr; 2 Laboratory for Medical and Veterinary Entomology/Centre of Excellence: One Health—Climate Change and Vector-Borne Diseases (CEOH), Faculty of Agriculture, University of Novi Sad, Trg Dositeja Obradovića 8, Novi Sad 21000, Serbia; 3 Vector and Pest Control Unit, Department of Epidemiology, Andrija, Štampar Teaching Institute of Public Health, Mirogojska c. 16, 10000 Zagreb, Croatia; suncica.petrinic@stampar.hr

**Keywords:** invasive species, *Aedes albopictus*, *Aedes japonicus*, mosquitoes in Croatia, vectors

## Abstract

*Aedes albopictus* and *Aedes japonicus* are invasive mosquito species that are causing great public concern. *Aedes albopictus* have successfully spread in Croatia, established in both the coastal and continental parts of the country, while *Aedes japonicus* is invading temperate climate areas. The invasive *Aedes* species are particularly attracted to the black plastic water containers and flower vases in cemeteries where they oviposit their eggs. Therefore, monitoring of this species was carried out in 12 cemeteries in Zagreb, using ovitraps with masonite strips as a substrate for oviposition. The monitoring was carried out from 2017 to 2020. The traps were inspected and the substrate was replaced every two weeks. This study showed that these two invasive species were present and very abundant in the cemeteries. In the case of *Ae. albopictus*, a higher population density and a greater number of occupied cemeteries were detected. This species was identified in all 12 cemeteries. *Aedes albopictus* was identified as the dominant species at all study sites. The spread of *Ae. japonicus* increased during 2018 in comparison to the previous year. Although this species was present in approximately 9% of the ovitraps, the observed population remained consistent throughout the course of the study. The findings indicate that cemeteries can be considered as significant public health hotspots, as the invasive *Aedes* mosquitoes tend to develop and reproduce in such environments. Consequently, the mosquito population of these two invasive species may only be reduced by applying integrated mosquito management measures, focused on the education of citizens.

## 1. Introduction

Invasive mosquito species have been successfully spreading in Europe for over four decades. One such species is the Asian tiger mosquito, *Aedes albopictus* (Skuse 1894), which has successfully invaded tropical, subtropical, and temperate regions of all continents, apart from Antarctica. Since its initial detection in Europe in 1979 in Albania [1], the range of *Ae. albopictus* has expanded significantly, with populations now established in over 25 countries across the continent [2]. The ongoing effects of climate change are facilitating the spread of this invasive species, with projections indicating a continued broadening of its range in both latitude and longitude [3,4,5]. Despite its tropical origin, the species has demonstrated remarkable adaptability, enabling rapid spread and adaptation to colder temperate zones. In the central Italian region, winter breeding has been observed in numerous locations [6]. Climate models predict that climate change, in conjunction with socio-economic behavior, will facilitate the continuous oviposition of *Ae. albopictus* throughout the year in multiple regions of the Mediterranean basin by 2080 [6]. All invaded tropical and subtropical regions are currently experiencing continuous reproduction of *Ae. albopictus* without an overwintering period [7,8]. This could serve as a comparative model for the future worldwide. Furthermore, it is challenging to implement effective control measures due to the vast number of artificial aquatic habitats that provide ideal breeding sites for this species.

The first report of the Asian tiger mosquito in Croatia was in 2004, in the city of Zagreb [9]. In the following years, established *Ae. albopictus* populations were detected throughout the entire country, in numerous locations on the Croatian coast and islands, where it has become a dominant mosquito pest [10,11]. Over the past decade, large populations of the species have been detected annually in the city of Zagreb [12,13]. Despite the identification of 32 mosquito species within the city of Zagreb to date [13], *Ae. albopictus* is considered as the most common mosquito species during the summer months [12].

The global prevalence of this mosquito species, combined with the high vector competence of the Asian tiger mosquito for over 26 arboviruses, represents a significant public health concern [14,15]. In Europe, arboviruses such as dengue (DENV) and chikungunya (CHIKV) have been demonstrated to successfully transmit to humans [16,17,18,19,20]. Despite the absence of endemicity of these two arboviruses in Europe, the favorable environmental conditions have facilitated the transmission of the competent vector, resulting in the occurrence of local transmissions of the virus. This is evidenced by the sporadic events of dengue transmission since 2010 [2]. In 2023, 130 autochthonous transmissions in humans were reported in the EU (France, 45; Italy, 82; Spain, 3) [17]. This represents a significant increase from the previous year (2022), when 72 autochthonous cases were reported in France and Spain [17], indicating that the dengue outbreaks are expanding their range.

In Croatia, sporadic imported cases of CHIKV and ZIKV infection have been continuously notified in travelers [21,22,23]. The first case of autochthonous dengue fever in Croatia was detected in 2010, following the return of a German tourist from a vacation in Croatia [24]. Following the notification of a dengue case from Germany, the implemented measures ultimately led to the diagnosis of a second autochthonous dengue fever case in a resident of the Pelješac peninsula (South Dalmatia). This area is the same one where the German patient had stayed, in which also 15 individuals with serological evidence of recent dengue infection were detected [24].

Another *Aedes* invasive mosquito species present in Croatia is *Aedes japonicus* (Theobald 1901) [12,25], also referred as Asian Bush mosquito or Asian rock pool mosquito. In Europe, the species was first identified in France, where it was successfully eradicated [26], and in Belgium [27], where it has not been observed to spread [14]. The species was subsequently encountered and introduced to Switzerland in 2008, where it subsequently expanded its range in all directions. The most recent report, based on the current known distribution as reported by the European Centre for Disease Prevention and Control (ECDC) [2] in October, indicates that the species is present in at least 15 European countries. In Italy, *Ae. japonicus* was first reported in 2015, in the most north-eastern provinces of the country, near the Austrian and Slovenian borders [28].

The first record of *Ae. japonicus* in Croatia was in Đurmanec, Krapina-Zagorje County (north-western Croatia) in 2013. Two years later, the species was identified in the city of Zagreb [25]. The species was subjected to annual monitoring and exhibited a wide distribution in temperate climate areas [29].

The species can tolerate a wide range of lower water temperatures but is absent in warm waters [30,31]. The areas in Croatia that are invaded by this mosquito species are characterized by a moderate climate. Thus, the species is present in Central Europe where it can be found from lowland to mountainous areas [32,33,34].

*Aedes japonicus* is not considered an important vector in the field experiments but has been found to be infected with West Nile virus (WNV), Japanese encephalitis virus, Cache Valley virus and La Crosse virus [35,36,37,38].

Both invasive *Aedes* species have adapted to the climate and environment of the city of Zagreb, continuously developing their population, especially in cemeteries. Although in their countries of origin both species develop in natural breeding sites, in temperate climates, they prefer artificial breeding sites such as water containers and catch basins. Invasive *Aedes* species are considered urban mosquitoes. Cemeteries are frequently situated in urban environments and function as preferred oviposition sites for mosquito vector species due to the availability of natural resources that are commonly present in most cemeteries [39].

In Croatia, the regulation of mosquito control is governed by the Law on the Protection of the Population against Infectious Diseases. Mosquito control is conducted at the local level, with the responsibility of organization and financing falling upon the local governmental units (counties). Each county’s Institute of Public Health is tasked with the preparation of mosquito control programs for their respective county. Croatia is constituted of 21 counties. Mosquito control is conducted by either the Institutes of Public Health or private companies. The surveillance of invasive mosquito species is conducted at the national level by the local Institutes of Public Health, with the resulting data being forwarded to the Croatian Institute of Public Health.

Since 1931, the city of Zagreb has employed organized mosquito control measures. At that time, the increased number of malaria patients constituted a significant trigger for the city administration to initiate a systematic program of indoor and outdoor mosquito control [40]. The current mosquito control program is in operation from March to the end of the year. It employs an integrated mosquito control approach, comprising the detection and removal of mosquito breeding sites, the use of larvicides in natural aquatic breeding sites and large water bodies (such as marshes and swamps), field inspections, the dissemination of information to the public and the implementation of larvicidal measures at the properties of citizens who have expressed concerns about mosquitoes and in the vicinity of their properties. Adulticide is used in response to an emergence of mosquito-borne diseases and when the mosquito population reaches a high nuisance level.

The objective of the study was to determine the abundance and seasonality of *Ae. albopictus* and *Ae. japonicus* in Zagreb cemeteries, thereby gaining a more comprehensive understanding of the two invasive species and their biology and preferences in the Croatian capital. Additionally, the study aimed to investigate the potential impact of different micro-locations on the coexistence of invasive mosquito species.

## 2. Materials and Methods

### 2.1. Study Area

The study area included the Croatian capital, the city of Zagreb (GPS coordinates 45.81250, 15.97778), and covers an area of 641 km^2^. A major part of Zagreb is located at an altitude of 112 m. The city is rich in landscape diversity; hills alternate with lowland landscape. The central part of the city is a densely populated urban area while the northern parts, situated on the Medvednica Mountains, are characterized by forest vegetation and smaller urban settlements. The majority of urban lowland areas are located along the Sava River valley. Agricultural areas are mainly situated in the outer north-eastern, eastern, and southern parts of the city. The city area abounds in the water surface. Numerous streams, seven artificial lakes and several artificial watercourses are in the city area.

**Figure 1:**
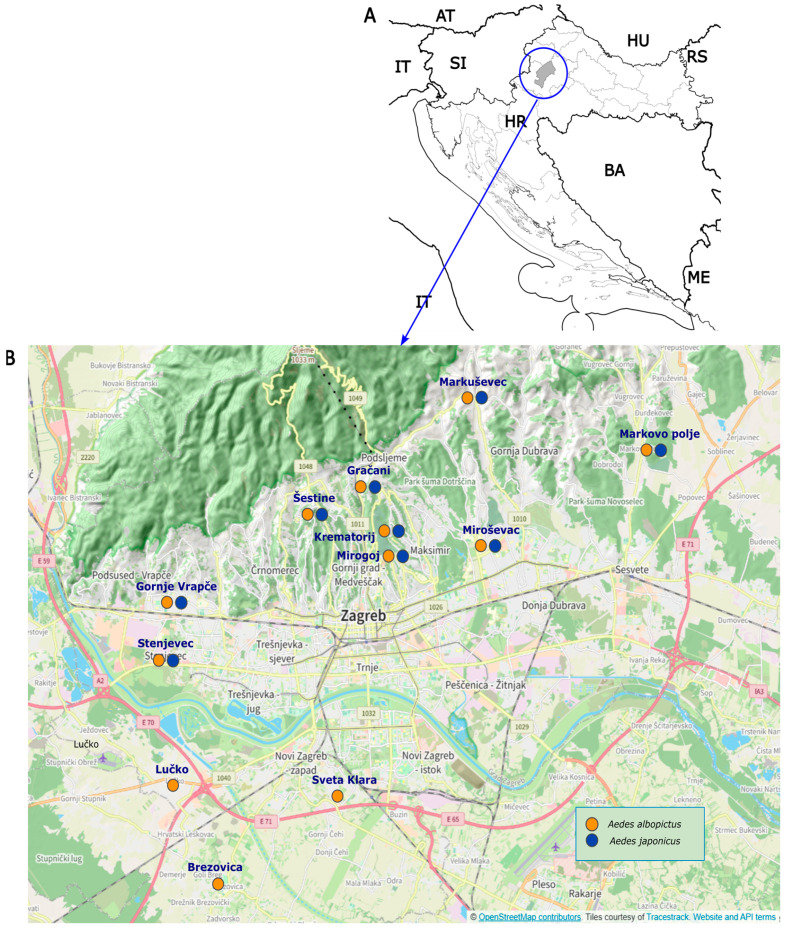
(**A**) The position of Zagreb city. Legend: HR—Croatia, IT—Italy, AT—Austria, BA—Bosnia and Herzegovina, HU—Hungary, ME—Montenegro, SI—Slovenia, RS—Republic of Serbia. (Created using: https://commons.wikimedia.org/wiki/File:Croatian_Counties,_Plain_Map_Colored.svg, accessed on 7 August 2024). (**B**) Distribution of *Aedes albopictus* and *Aedes japonicus* in cemeteries of Zagreb during 2018 (Created at: https://www.openstreetmap.org/search?query=Zagreb#map), accessed on 7 August 2024.

There are total of 25 cemeteries in the Zagreb area, 17 being in the northern parts and eight in the southern parts of the city. This research involved 12 of them to monitor the invasive mosquito species. Out of these 12 cemeteries, nine were in the northern and three in the southern areas of the city (Table 1, Figure 1). *Aedes japonicus* disseminated throughout the entire Krapina-Zagorje county by the end of 2015, which is located north-western of the city of Zagreb. Additionally, the initial record of *Ae. japonicus* in Zagreb occurred in August 2015 at two locations on the northern periphery [12,25]. It was therefore anticipated that the species would spread to the area of the city of Zagreb from the north [12,13,25]. Considering that the species started its spread from the northern side of the city, more cemeteries were selected in the north. Two additional criteria were respected in the selection: cemeteries should be evenly distributed, covering the whole city of Zagreb, and that the entrance was enabled during the working hours of the technicians who sampled the invasive mosquitoes.

The Zagreb climate is characterized as a moderate continental climate, with hot summers and without extremely dry periods. The average daily temperature during the summer months (June to August) varies between 20 and 25 °C. The temperature is the highest in July. The average temperature of the coolest month (January) is above 0 °C. The mean annual precipitation is 800–1000 mm, with a minimum in winter (approximately 40 mm in February) and a maximum in summer (approximately 100 mm in August).

### 2.2. Oviposition Monitoring

The study was conducted during a four-year period, 2017–2020. The surveillance was conducted at 12 locations (12 cemeteries) with three ovitraps per location (a total of 36 ovitraps). The distance between the traps in cemeteries was approximately 50 m, placed either on the ground or up to 50 cm above the ground, hidden by vegetation in shaded and wind-protected places. The ovitrapping was continuously conducted from the 17th week (second half of April) to the 46th week (middle of November) in each of the research years. A total of 2160 ovitraps were set in the four-year period, 540 each year.

The ovitraps consisted of a 500 mL volume black plastic container (15 cm high and 11.5 cm in diameter), filled with tap water (approximately 250 mL) and a wooden (Masonite) strip (16 × 2.5 × 0.4 cm) as an oviposition substrate. A hole was drilled 2.5 cm below the edge of the ovitrap to prevent the water from overflowing after the rains.

The inspection of the ovitraps was carried out bi-weekly and then the wooden strips and the water from the ovitraps were replaced. Each strip from the ovitraps was placed in individual paper towels. The containers were emptied, rinsed, and the inner surface carefully cleaned to remove any remaining eggs. The strips were delivered to the Medical Entomology Laboratory, Vector and Pest Control Unit, Department of Epidemiology, Andrija Štampar Teaching Institute of Public Health in Zagreb. During the four-year period, a total of 2110 wooden strips were found in ovitraps (2017: 536; 2018: 537; 2019: 521; 2020: 516), while 34 strips and 16 ovitraps with strips were missing.

### 2.3. Laboratory Work

The eggs (unhatched and hatched) were counted with a stereomicroscope (80× magnification). The total number of eggs per ovitrap was separately recorded for the *Aedes* invasive species and *Aedes geniculatus*. The eggs of the invasive mosquitoes, *Ae. albopictus* and *Ae. japonicus*, cannot be accurately distinguished under a stereomicroscope, whereas eggs of *Ae. geniculatus* are easy to distinguish and count.

To accurately determine the presence of an invasive species that oviposited on the strip (*Ae. albopictus* or *Ae. japonicus*) and to avoid confusion with other mosquito species that also lay their eggs in small water containers, all eggs were hatched. The larvae were hatched and reared using the same type of ovitrap. The procedure was carried out in the laboratory conditions at an air temperature of 22–27 °C, a relative humidity of 50–70% and in daylight exposure. The larvae were reared to the L4 larval instar, then stored in 96% ethanol until identification. Larvae were morphologically identified using the identification keys [41,42]. In the containers, where both invasive species were found on the strip, the larvae of the *Aedes* species were not counted, but this trap was recorded as a separate category, named gathered *Ae. albopictus* and *Ae. japonicus*.

### 2.4. Data Analysis

Meteorological data (precipitation and temperature) were obtained from a Croatian meteorological and hydrological station, located at Zagreb-Maksimir, Zagreb.

To estimate the distribution, seasonal abundance and activity of the invasive *Aedes* mosquitoes, the following parameters were counted:

1. Positive ovitrap index (POI): the percentage of positive ovitraps out of the total number of ovitraps inspected per bi-weekly sampling.
2. Mean eggs per trap (MET): the mean number of eggs per ovitrap/number of positive ovitraps.
3. Based on the results obtained, species association indices (AIs) were calculated according to Dice [43], which are based on the occurrence of species at the ovitrap site. Species association indices quantify the proportion of sites where two species occur in combination compared to the total number of sites where one of the corresponding species is found. The degree of overlap between the biotic and abiotic structure requirements of the collection sites can be estimated using the species association index. In general, more abundant and ubiquitous species are more frequently associated with other species [43]. The association index is given a value of zero if several species occur in the cemetery, but these species did not share a common oviposition site (were not found in the same ovitrap). Index 1 indicates that one species was always related to another.
4. The total number of eggs per sample was accurately counted. In the samples where more than one species was present, the exact number of eggs per species was not determined (eggs of *Ae. albopictus* and *Ae. japonicus* cannot precisely be differentiated). The aim was to analyze the distribution of species (presence/absence), and not the abundance. Therefore, only those cemeteries where only one species was present during the entire monitoring period can be statistically analyzed. Three cemeteries where only *Ae. albopictus* was present were selected for further statistical comparison: Brezovica, Lučko and Sveta Klara. The number of eggs per day at each cemetery was calculated and compared to determine the differences between the *Ae. albopictus* populations at the sites (cemeteries), but also to compare changes in the populations over four years.

Data were analyzed using Statistica v.14.0.0.15 (1984–2020 TIBCO Software Inc., Palo Alto, CA, USA). The number of eggs per day was compared using the general linear model (GLM), repeated measurements, comparing the mean values of eggs using the multivariate test (Pillai’s test) with a significance level *p* < 0.05. Three ovitraps per cemetery served as three replicates. When the number of eggs was compared within one cemetery in different years, the dependent variables were eggs per day in different weeks of sampling and the categorical factor was years. When we compared the number of eggs in different cemeteries but for the same year, the dependent variable was the same, but the categorical factor was the cemetery.

We compared egg numbers per day between the cemeteries, but also the number of eggs on the same cemetery in the different years. Scatterplots of eggs per day was represented against weeks, categorized by cemetery. To present the comparison of the eggs in different years in the same cemetery, a scatterplot of eggs per day against weeks categorized by year was created for each of the three cemeteries. In the graphs, x represents weeks and y represents number of eggs per day.

## 3. Results

### 3.1. Meteorological Conditions

The recorded differences in the meteorological conditions (particularly temperature) during the four years of mosquito monitoring (2017–2020) might have caused differences in the population dynamics. The difference was found at the beginning of the year. The average daily temperature in the four years in January ranged from −3.2 °C in 2017 to 5.2 in 2018; January was warmer in 2018 compared to the other years. Despite the temperature drop in February of that year, in April the mean monthly temperature was already approximately 3 °C higher than in April during the three other observed years (Figure S1). The warmest month of the years was August when the average daily temperatures during the four years were similar, with a maximum 23.7 °C in 2017 and 2018. The total amount of precipitation from 2017 to 2020 was as follows: 897 mm, 853.6 mm, 1000.5 mm and 950.4 mm (Figure S2). The relative humidity in these four years averaged between 59.75 and 83.5%. The lowest mean humidity in the individual years was not below 50% and not above 87% (Figure S3).

The total amount of precipitation from 2017 to 2020 was as follows: 897 mm, 853.6 mm, 1000.5 mm and 950.4 mm. In 2018, the double amount of precipitation was recorded in February and March compared to the precipitation in the same months during the other observed years. The highest amount was recorded in 2017 in September, when 239.6 mm of rain fell (Figure S2). The relative humidity in these four years averaged between 59.75 and 83.5%. The lowest mean humidity in the individual years was not below 50% and not above 87% (Figure S3). In 2018, the average monthly relative humidity in February and March was higher than in other observed years.

### 3.2. Seasonal Activity of Aedes Invasive Species, Positive Ovitrap Index (POI) and Mean Eggs Per Trap (MET)

During the four-year period of monitoring invasive *Aedes* species in 12 cemeteries, a total of 2110 ovitraps (Masonite strips for oviposition) were inspected. Two invasive (*Ae*. *albopictus* and *Ae. japonicus*) and one native (*Ae. geniculatus*) mosquito species were identified from the eggs collected in the ovitraps. The species *Ae. albopictus* and *Ae. japonicus* were present in a sum of 1218 ovitraps, i.e., 57.73% of the ovitraps were positive, which means that at least one of these two species was present in the trap. During the four-year monitoring, 135,830 mosquito eggs were counted. In ovitraps with only *Ae. albopictus* eggs (884; 72.58% positive ovitraps), a total of 109,969 eggs were recorded, which represent 80.96% of the total mosquito eggs. Ovitraps with only *Ae. japonicus* eggs (35; 2.87% positive ovitraps) collected 3350 eggs, or 2.46% of total mosquito eggs. Both invasive *Aedes* species were detected in 72 (5.91% positive ovitraps), with 13,326 eggs, or 9.81% of total mosquito eggs.

*Aedes albopictus* was present in all 12 cemeteries with population activity ranging from April to November. In the case of *Ae. japonicus*, eggs were found in six to nine cemeteries, depending on the year. The Asian bush mosquito population showed different oviposition activity in the four years of monitoring. In 2017, eggs were found from April to August. In the following two years (2018 and 2019), the population was active slightly later, from early June to early September. In 2018, however, the eggs were once again found in one ovitrap in mid-October. In 2020, egg laying began in mid-May and ended in early October.

**Figure 2:**
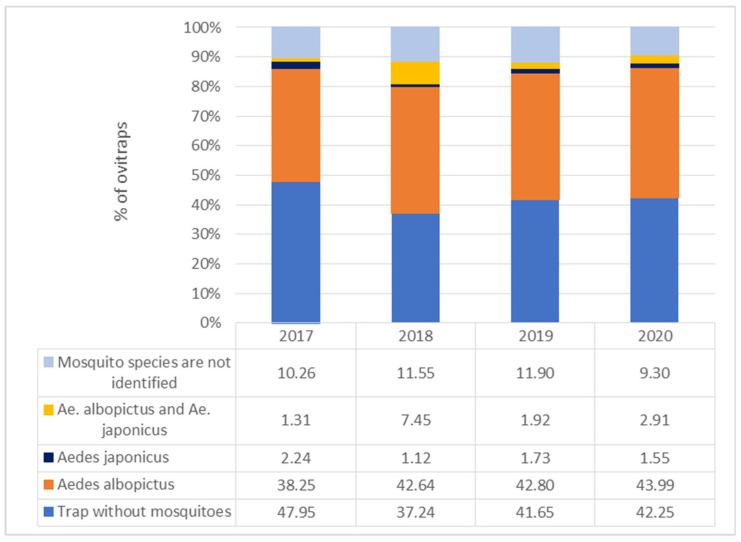
Percentage of ovitraps in which mosquito species were detected in a four-year period of *Aedes* invasive mosquitoes monitoring, according to the criteria present/absent from 2017 to 2020 in cemeteries of Zagreb.

Observing the entire study period (2017–2020), the lowest percentage of positive traps was recorded in 2017 (52.05%). In 2017, a total of 279 ovitraps were positive for the *Aedes* invasive species. A total of 26,273 eggs were counted in 12 cemeteries. The highest number of positive ovitraps was recorded in 2018 (337 ovitraps; 62.76%) with 51,995 eggs counted. The other two years (2019 and 2020) had similar percentages of positive traps (2019: 58.35%; 2020: 57.75%) and mosquito eggs (2019: 27,889 eggs; 2020: 29,673 eggs). The dominant species in the majority of analyzed ovitraps was *Ae. albopictus* (Figure 2)*. Aedes albopictus* was the only species present in most of the positive ovitraps without other species, with percentage values ranging from 38.25% in 2017 up to 43.99% in 2020. *Aedes japonicus* was more frequently detected in the ovitraps together with *Ae. albopictus* then as a single species.

The species *Ae. japonicus* was recorded in a very low percentage of ovitraps (between 1.12% in 2018 and 2.24% in 2017) alone without cohabitation. Two invasive mosquito species, *Ae. japonicus* and *Ae. albopictus*, did not demonstrate a conspicuous pattern of cohabitation throughout the four-year period, showing very diverse results in these years, i.e., 1.31% in 2017 and 7.45% in 2018. In the subsequent years after 2018, the species showed decreased cohabitation, 1.92% in 2019 and 2.91% in 2020.

**Figure 3:**
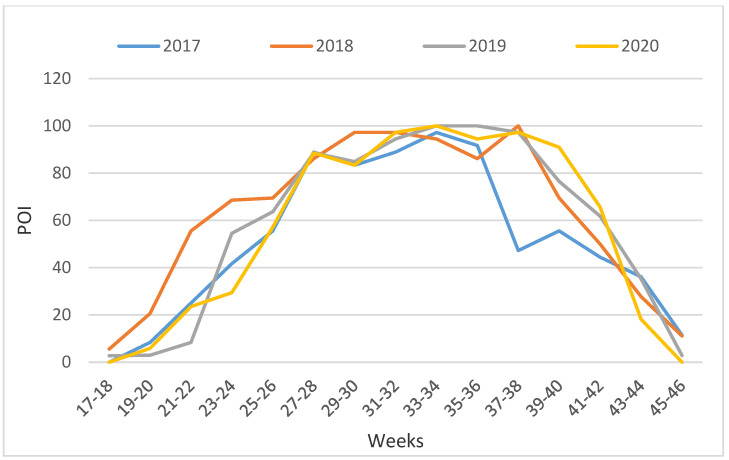
Positive Ovitrap Index (POI) of *Ae. albopictus* and *Ae. japonicus* from 2017 to 2020 in 12 cemeteries in Zagreb.

The distribution of *Ae. albopictus* and *Ae. japonicus*, expressed as the positive ovitrap index (POI), in the 12 cemeteries is presented in Figure 3. Data analysis showed that the POI was highest in the 33rd and 34th week of monitoring (second half of August) which was evident in all years except 2018 when the peak of activity was in the 37th to 38th weeks (mid-September). Additionally, in 2018, the POI was significantly higher in the spring, from the 19th to 24th weeks, than in other years.

**Figure 4:**
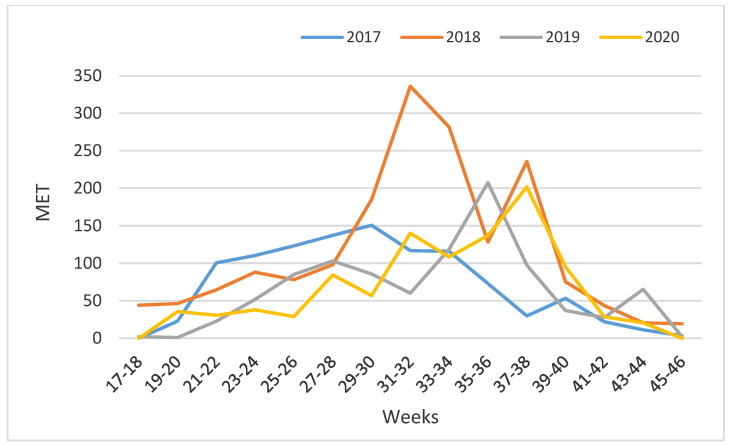
Mean Eggs Per Trap (MET) of *Ae. albopictus* and *Ae. japonicus* from 2017 to 2020 in 12 cemeteries in Zagreb.

The average number of eggs per trap (MET) over a four-year period varied between zero (no eggs were recorded in three samplings) and 336 (a peak of activity in 2018). Analyzing the MET, it is noticeable that 2017, 2019 and 2020 were similar (Figure 4). More eggs were collected in 2018 compared to the other three years.

Oviposition activity began in April (17–18 weeks) in 2018 and 2019 with 5.56% and 2.78% positive traps, respectively. The season of oviposition activity was completed in November (week 45–46) in 2017, with 11.43% positive ovitraps, and in 2018 and 2019 with 11.11% and 2.86% positive ovitraps, respectively. The dynamics of oviposition are shown in Figure S4, where the green color represents the samples with the lowest values and the red color the highest values for the total number of eggs, POI and MET.

**Figure 5:**
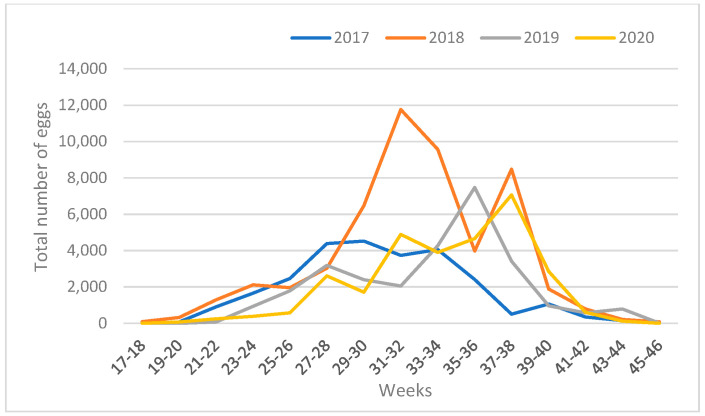
Total number of eggs per sampling of *Ae. albopictus* and *Ae. japonicus* from 2017 to 2020 in 12 cemeteries in Zagreb.

The total number of eggs per sampling (for two weeks) in all 12 cemeteries ranged from 0 to 11,761 (31–32 weeks in 2018). A very high population was reported from the beginning of July to mid-September (Figure 5). The highest number of eggs for one year was sampled in 2018, a total 51,995; while a total number of eggs during the other three years ranged from 26,273 in 2017 to 29,673 in 2020. For four years, a total of 135,830 eggs were counted from the ovitraps, of which 80.96% were eggs from traps with *Ae. albopictus* eggs only, and 2.47% of eggs from traps with the *Ae. japonicus*.

### 3.3. Comparison of the Cemeteries and Association Indices of the Aedes Species

The investigated cemeteries in Zagreb were particularly suitable for invasive *Aedes* species (Table 2), but also for the native *Ae. geniculatus*. The cemeteries with the highest number of invasive mosquito species eggs in the period 2017–2020 were Stenjevec with an average number of invasive species eggs during one year of 5229 ± 2733 eggs and Miroševac with 4076 ± 1881 eggs. The highest total number of eggs per cemetery per year was counted in Stenjevec, with 9292 eggs in 2018.

The lowest average number of eggs was recorded in Brezovica (1636 ± 425) and Markuševec (1729 ± 1072).

The highest total number of eggs collected in the four-year period was recorded in Stenjevec (20,916 eggs) and the lowest total number in Brezovica (6544 eggs).

Although the number of eggs collected per year varied among the cemeteries, the portion of eggs in the total number of eggs per year did not vary greatly between the 12 cemeteries. The percentages ranged from 2% (Brezovica in 2018) to 18% (Stenjevec in 2018).

The association indices are presented vertically in Table 3. The analyses showed that in three cemeteries (Lučko, Brezovica and Sveta Klara) during four years of monitoring, (2017–2020) *Ae. japonicus* was not detected.

The index had a value of zero six times in four years, indicating that both species were present in the same cemetery but did not share ovitraps. This occurred in three cemeteries in 2017, two cemeteries in 2019 and only once in 2020.

*Aedes albopictus* was present and abundant in all four years in all cemeteries. *Aedes albopictus* and *Ae. japonicus* were found in the same trap 72 times (eggs of both species in the same trap) in four years in twelve cemeteries. During 2018, these species invaded eight cemeteries at the same time. *Aedes geniculatus* was in the same trap during the four years with *Ae. albopictus* 37 times (26 times only in 2020) and with *Ae. japonicus* 10. All three species were together in the same traps only nine times.

In the first year (2017), the indices of *Ae. japonicus* fluctuated between 0 and 0.67, (mean was 0.24 and median 0.19) indicating that the species does not share the oviposition site with *Ae. albopictus* so frequently (Table 3 and Figure S5). In the following year (2018), *Ae. japonicus* was strongly associated with *Ae. albopictus* (the AI was between 0.67 and 1, mean was 0.91 and median 0.96). In all cemeteries that were positive for the presence of *Ae. japonicus*, the species was associated with *Ae. albopictus* in at least one ovitrap. In the last two years (2019 and 2020), *Ae. japonicus* was less associated with *Ae albopictus* (SAIs in 2019 and 2020 were between 0 and 1 depending on the cemetery). The mean in 2019 was 0.61 and the median 0.67; in 2020, the mean was 0.65 and median 0.84.

Considering the eggs of native species, *Ae. geniculatus* was detected in ovitraps. In the first year of monitoring, this species was not found in any of the twelve cemeteries. In the following three years, *Ae. geniculatus* was present in some of the selected cemeteries (Table 3) and the number of eggs on wooden strips ranged from 3 to 195. The Species Association Index showed that association of *Ae. geniculatus* and *Ae. albopictus* in 2018 was 1 in all positive cemeteries on the presence of *Ae. geniculatus* (mean and median were 1), meaning that this native species was in all traps gathered with *Ae. albopictus*, but in the same year it was less associated with *Ae. japonicus* (SAI ranged from 0 to 0.5, with mean and median 0.25).

The following year was different with *Ae. geniculatus.* The species was less associated with both *Ae. albopictus* and *Ae. japonicus* than the previous year (SAI with *Ae. albopictus* ranged between 0.67 and 1, mean was 0.78 and median 0.67, and SAI with *Ae. japonicus* was 0 to 0.33 with mean 0.11 and median 0).

During 2020, the species was present in eight out of twelve cemeteries. According to the association index, *Ae. geniculatus* was more associated with *Ae. albopictus* (SAI ranged from 0.88 to 1, mean was 0.97, median was 1) than with *Ae. japonicus* (SAI ranged from 0 to 1, mean was 0.42, median was 0.38)*,* which could be explained by the larger and more widespread population of the Asian tiger mosquito compared to the other one. An overview of the association of the three species over four years (2017–2020) can be found in Figure S5 and Table 3.

In the Figure S5, colored cells show the presence and co-habitations of three analyzed species. During all four years, the dominant species was *Ae. albopictus*. The number of positive traps in 12 cemeteries (Table 3) for the presence on *Ae. albopictus* was highest in 2018 (total 269 traps, min 14 in Brezovica, max 33 in Stenjevec). *Aedes albopictus* was present in 214 traps during 2017 (min. 10 in Markuševec, max. 24 in Stenjevec), in 230 traps during 2019 (min. 13 in Šestine, max. 27 in Miroševec) and in 242 traps during 2020 (min. 12 in Markovo Polje, max. 25 in Lučko).

Presence of the *Ae. japonicus* was most frequent in 2018 when 46 traps were positive in 12 cemeteries (species was not present in four cemeteries, max. was 11 in Markovo Polje). This species was reported in 19 traps during 2017 (not present in five cemeteries, max. was eight in Markovo Polje), in 19 traps during 2019 (no *Ae. japonicus* in three cemeteries, max. was three traps per cemetery in Gračani, Markuševec and Šestine). In 2020 this invasive mosquito was present in 23 traps (in four cemeteries, species was not reported, max. eight in Markovo Polje).

*Aedes geniculatus* was not reported in 2017 in any of the cemeteries. This species was most frequently found in 2020 when it was present in 28 traps. During this year, four cemeteries were not selected by *Ae. geniculatus*, and the max. positive traps was 10 in Mirogoj. Summarizing all four years, the *Ae. albopictus* was present in 955 samples (traps), *Ae. japonicus* in 107 and *Ae. geniculatus* in 41. Although we could conclude that the *Ae. albopictus* was very well dispersed in all cemeteries for four years, the highest number of positive samples was in Stenjevec (105 positive traps), then in Miroševac (102) and Gračani (92 traps). We could not say that this species preferred northern compared to southern parts because two of these cemeteries are on the north and one was on the south part of the city.

The situation was different with *Ae. japonicus* which did not disperse so quickly as *Ae. albopictus. Aedes japonicus* preferred Markovo Polje (29 positive traps) where two times more samples were positive compared to the second-most-inhabited cemetery (Gračani and Markuševec). Our finding demonstrated that *Ae. japonicus* was most present in the north-eastern part of the city and it is populating areas towards the western part of Zagreb.

*Aedes geniculatus* was sporadically present in seven cemeteries, while in three it was not present at all. Two cemeteries were most inhabited by this native species, and those were Mirogoj (15 positive traps) and Krematorij (14 positive traps), both located in the northern side of the city.

**Figure 6:**
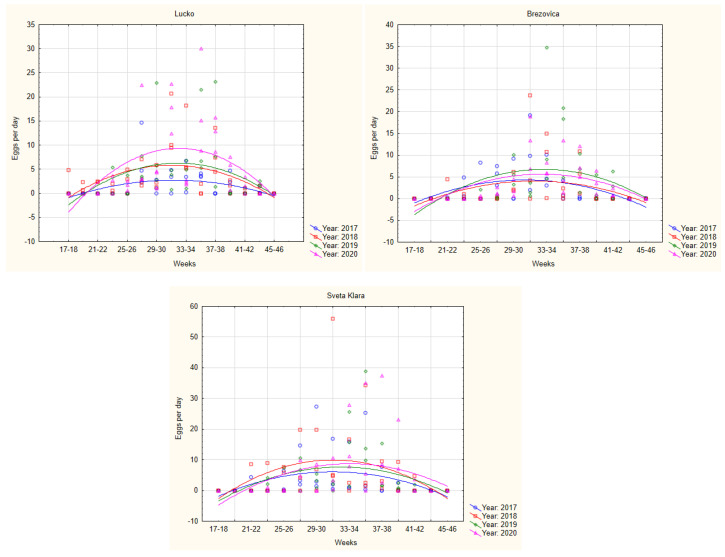
Number of *Ae. albopictus* eggs per day in cemeteries Lučko, Brezovica and Sveta Klara in four years of *Ae. albopictus* monitoring (2017–2020). Comparison of eggs in different years sampled in each separated cemetery.

**Figure 7:**
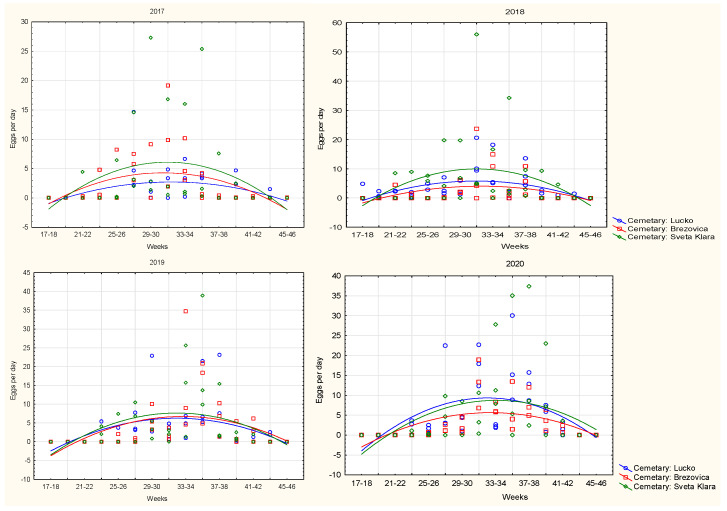
Number of *Ae. albopictus* eggs per day in cemeteries Lučko, Brezovica and Sveta Klara in 2017–2020. Comparison of eggs in cemeteries collected in each separated year.

The number of *Ae. albopictus* eggs per day collected from the cemeteries are shown in Figure 6 and Figure 7. Figure 6 shows a comparison of eggs in the same cemetery over four years, and Figure 7 shows a comparison of egg number in all three cemeteries, separately for each of the four years. In the scatterplot, it is presented that the population of *Ae. albopictus* was similar to Brezovica and Sveta Klara in all four years. In Lučko, however, the population in 2020 was higher during the peak of the season.

Regardless of the different number of eggs collected in each cemetery over four years, there was no significant difference (Lučko 2017–2020: 312.03, df = 3, MS = 104.01, F = 1.83, *p* = 0.22; Brezovica 2017–2020: SS = 77.3, df = 3, MS = 25.76, F = 0.72, *p* = 0.57 and Sveta Klara 2017–2020: SS = 147.85, df = 3, MS = 49.29, F = 0.11, *p* = 0.95).

When the cemeteries were compared for each individual year, the statistical comparison showed that the number of eggs collected did not differ significantly in any of the four years (Lučko vs. Brezovica vs. Sveta Klara in 2017: SS = 69.08, df = 2, MS = 34.5, F = 0.3 *p* = 0.75; Lučko vs. Brezovica vs. Sveta Klara in 2018: SS = 263.5, df = 2, MS = 131.8, F = 0.44, *p* = 0.66; Lučko vs. Brezovica vs. Sveta Klara in 2019: SS = 11.88, df = 2, MS = 5.94, F = 0.06, *p* = 0.94; Lučko vs. Brezovica vs. Sveta Klara in 2020: SS = 161.28, df = 2, MS = 80.6, F= 0.35, *p* = 0.72).

## 4. Discussion

Since the detection of two invasive *Aedes* species in Croatia, *Ae. albopictus* in 2004 [9] and *Ae. japonicus* in 2013 [25], the populations of the species continue to rise every year. The city of Zagreb has reflected the high environmental suitability for the reproduction and dispersal of both mosquito species.

Over a four-year period, 135,830 eggs were collected from 12 cemeteries in Zagreb. Populations of all species fluctuated in the selected period (2017–2020) due to different abiotic factors (air temperature, relative humidity and precipitation).

In our study, two cemeteries, Stenjevec and Miroševac, were with the highest number of *Aedes* invasive mosquito eggs. In contrast, Markuševec and Brezovica had the lowest number of eggs in four years.

The oviposition season started early in Zagreb. The first eggs were laid in April (17th–18th weeks after the beginning of the year). Regardless of the continental climate in Zagreb, same as in Novi Sad, Serbia, the beginning of oviposition activity in Novi Sad was different and started several weeks later (23rd–24th week) [44]. Temperature is considered the most determinant predictor of *Ae. albopictus* oviposition and its population growth in the Balkan countries [45], which influences the beginning of oviposition activity of these species [46]. It is evident that the temperature in Zagreb during the four-year study period had an influence on the *Ae. albopictus* populations. The population was the highest in 2018. The hypothesis is that due to the warmer beginning of the year, this species was developing faster. All mentioned above, the mean monthly temperature in April was approximately 3 °C higher than in April during the three other observed years.

In the cemeteries of Zagreb, the oviposition activity peak differentiates in the four analyzed years. The mean number of laid eggs was from 29 to 36 weeks depending on the year which corroborates with the results given by Zitko and Merdić [11] for Split. Their study showed that the peak of oviposition activity in Split was between week 28 and week 35. Comparable results were demonstrated by neighboring country, northern part of Italy, where the Asian tiger mosquito reached the oviposition peak between week 27 and week 37 [47].

The oviposition activity in the cemeteries of Zagreb ended in November, which was similar to Novi Sad [44]. However, previous studies in Croatia have shown that the oviposition season (until early December) lasted longer in the town Split, located in the south of Croatia, where the climate is Mediterranean [11], opposed to Zagreb with the continental climate.

Long oviposition periods (April–November, 2017–2020) resulted in a high number of eggs laid each year in each of the 12 selected cemeteries in Zagreb. Based on the total number of eggs collected, the peak of oviposition activity of invasive *Aedes* mosquito species in Zagreb cemeteries was mainly in August (33rd–34th week), with the exception of 2018, when the peak was in September.

When analyzing the cohabitation of three species (*Ae. albopictus*, *Ae. japonicus* and *Ae. geniculatus*), no specific pattern of their association was found.

When we compared the number of eggs in the cemeteries where only the *Ae. albopictus* was present, no preference was found for the cemetery.

The Asian tiger mosquito was a dominant species in all cemeteries. In three cemeteries (Lučko, Brezovica and Sveta Klara), *Ae. albopictus* occurred as a single species.

The possible explanations for the dominance of *Ae. albopictus* in urban areas were presented in the previous studies. Dissimilar environmental conditions are suitable for these two mosquito species. *Aedes albopictus* showed a preference for urban and suburban areas, whereas the *Ae. japonicus* prefers rocky areas (rock pools), vegetated areas and forests, and is therefore more common in rural, suburban and agricultural areas [48,49,50,51]. *Aedes japonicus* is primarily distributed in geographically colder and temperate climates [50]. Hot and dry summer conditions in urban areas may have a negative impact on the distribution of *Ae. japonicus*, while exactly such conditions are more favorable for the increase of the population of *Ae. albopictus* [52,53]. Our research demonstrated that *Ae. japonicus* was present in nine cemeteries in the northern part of the city and was not found in three cemeteries in the southern urban part of the city. The results confirmed our hypothesis that the species will first spread and become established in the northern parts, which are closer to Krapina-Zagorje County and situated on the Medvednica Mountains characterized by forest vegetation and smaller urban settlements.

Other authors claim that the successful invasion of *Ae. albopictus* is partly due to its negative impact on native species, as it is considered a superior competitor in larval habitat and is capable of mating interference and satyrization of *Aedes aegypti* [54,55]. Factors that may have an impact on *Ae. albopictus* competition with *Ae. aegypti* include microclimate [56], detritus resources [57], parasitism [58] and predation [59]. Although it is shown that *Ae. albopictus* may behave as the superior competitor [33,58,60,61,62], the authors Westby et al. [63] do not consider that as a rule for this species but claim that dominance is rather complex and depends on other factors. Analyzing these two invasive *Aedes* species in the earlier studies, it was assumed that the selected monitoring area and the type of breeding sites could be related to the presence and abundance of these two species.

The authors Cevidanes et al. [64] showed that the probability of finding *Ae. japonicus* was higher at gas stations and in industrial areas (both places are usually surrounded by shrubs and vegetation-rich areas and have a substantial number of artificial breeding containers). Their results demonstrated that the probability of finding *Ae. albopictus* is higher in parking lots, which could be due to the passive dispersal of this species via cars and other vehicles [65,66].

The study by Cevidanes et al. [64] showed that *Ae. albopictus* was more abundant in communities with a higher population (human) density (urban environment), while *Ae. japonicus* preferred communities with lower population density, such as peri-urban and rural environments [67]. Although *Ae. japonicus* may occasionally occur in urban areas, such as in cemeteries in our study, hot and dry summer conditions have a negative impact on its life cycle [68].

In addition, a greater diversity of mammalian hosts on which they can feed could be the reason they prefer peri-urban and rural areas. Although the two species appear to coexist without evidence of displacement, the possible competitive interaction between the larval stages of the two species should not be ignored and should be investigated further.

Analysis of the occurrence of invasive *Aedes* mosquitoes and the degree of urbanization (urban, suburban, peri-urban) revealed that, *Ae. albopictus* was 4.39 times more likely to be found in suburban areas than in peri-urban areas, while *Ae. japonicus* was more likely to be found in peri-urban areas. Furthermore, the presence of *Ae. albopictus* was significantly associated with municipalities with a higher population density (mean = 2983 inhabitants/km^2^), while *Ae. japonicus* was associated with a lower population density (mean = 1590 inhabitants/km^2^) [64].

Environmental change and urbanization have an indirect impact on the community of mosquito species, which can sometimes lead to a loss of biodiversity due to anthropogenic changes [69].

Monitoring of *Aedes* invasive species demonstrated the dominant population of *Ae. albopictus* opposed to other present mosquito species in cemeteries. This finding is in accordance with the study by Armistead et al. [61] who stated that as a consequence of higher survivorship, shorter developmental time and a significantly higher estimated population growth rate under conditions of interspecific competition, *Ae. albopictus* larvae exhibited a competitive advantage over *Ae. japonicus* in water-containing cups provided with oak leaves.

*Aedes japonicus* did not show any further invasion of the cemeteries, meaning that the population was not spread significantly over this four-year period. In the first year, *Ae. japonicus* was present in six cemeteries. This species spread to three new cemeteries in 2018. Afterward, the species was detected in eight cemeteries in the two following years (2019 and 2020). The number of positive ovitraps was also different in different years but without a specific trend: 19 positive ovitraps in 2017, 46 in 2018, 19 in 2019 and 23 in 2020. The hypothesis is that a significant increase of *Ae. japonicus* in the ovitraps during 2018 is caused by the mean April and May temperature; as we mentioned above, it was approximately 3 °C and 1.8 °C higher than in April and May during the three other observed years. Additionally, winter was very mild at the end of 2017 and at the beginning of 2018 (no minus of mean daily temperatures) compared to other observed years.

Based on the number of positive ovitraps, this species was present in approximately 9% of samples. *Aedes japonicus* particularly increased its population in cemeteries during 2018. It is incontestable that this species has become established in the northern parts of the city.

*Aedes albopictus* was present in 100% of observed cemeteries where the monitoring occurred. This species represents a very persistent nuisance of the urban environment and a noteworthy concern to the public health of Zagreb due to its vectorial competence for the pathogens causing outbreaks in Europe.

## 5. Conclusions

*Aedes albopictus* successfully invaded all 12 cemeteries in Zagreb indicating the suitable environment for this species in urban zones of the country. The population develops and reproduces from April until November, representing a nuisance almost the entire year in Zagreb.

*Aedes japonicus* did not demonstrate such aggressive invasion compared to *Ae. albopictus*, but the population that selected the cemeteries for its development was stable in this environment. This species was active in the cemeteries from May to September and in some cases until October.

Mosquito populations of these two invasive species may only be reduced by applying integrated mosquito management measures, focused on the education of citizens.

## Figures and Tables

**Table 1:** Cemeteries in which the invasive mosquito species were monitored (coordinates, elevation, and the area in ha).

Cemetery Name	GPS Coordinates	Elevation (m)	Cemetery Area (ha)
Markovo polje	45.86072, 16.12917	137	26.42
Miroševac	45.84278, 16.03961	171	42
Markuševec	45.87455, 16.01244	238	1.8
Gračani	45.85481, 15.97031	267	0.34
Krematorij	45.84582, 15.98263	249	20
Mirogoj	45.84549, 15.98146	250	72.4
Šestine	45.85341, 15.94994	289	1
Gornje Vrapče	45.8276, 15.90775	181	2.3
Stenjevec	45.81165, 15.88851	119	1.7
Lučko	45.76004, 15.88222	118	2
Brezovica	45.73277, 15.89588	123	1.5
Sveta Klara	45.75693, 15.9717	113	1.11

Coordinates and elevation taken from: https://www.google.com/intl/hr/maps/about/mymaps accessed on 30 October 2021.

**Table 2:** Total number of eggs of *Aedes* invasive species in 2017–2020 in 12 cemeteries of Zagreb.

Cemeteries	Years						
	2017	2018	2019	2020	Total	Mean	St Dev
Mirogoj	1380	2506	2365	2669	8920	2230	580
Krematorij	4096	2452	1587	2235	10,370	2592	1068
Gračani	2624	3982	2094	2465	11,165	2791	824
Markuševec	698	3205	1276	1740	6919	1729	1072
Miroševac	3540	6733	3732	2302	16,307	4076	1881
Markovo polje	2086	5101	2608	2561	12,356	3089	1362
Lučko	882	2019	2089	3069	8059	2014	894
Brezovica	1316	1265	2159	1804	6544	1636	425
Stenjevec	4218	9292	4029	3377	20,916	5229	2733
Gornje Vrapče	2146	7492	1657	2251	13,546	3386	2749
Šestine	1378	4701	1835	2292	10,206	2551	1481
Sveta Klara	1909	3247	2458	2908	10,522	2630	579
Total	26,273	51,995	27,889	29,673	135,830	33,957	12,105

**Table 3:** Species association indices (Dice 1945) based on the species occurrence in the cemeteries sampled by ovitraps.

Cemeteries		2017			2018				2019				2020		
		*Ae. alb*	*Ae. jap*	* *	*Ae. alb*	*Ae. jap*	*Ae. gen*	* *	*Ae. alb*	*Ae. jap*	*Ae. gen*	* *	*Ae. alb*	*Ae. jap*	*Ae. gen*
Mirogoj	Positive samples	12			20	5	2		16	2	3		17	1	10
	Oviposition index	1			1	0.25	0.10		0.94	0.12	0.18		0.94	0.06	0.56
	SAI	NA													
	*Ae. albopictus*					1	1.00			1	0.67			0	0.90
	*Ae. japonicus*				0.25		0.50		0.13		0.33		0		0.10
	*Ae. geniculatus*				0.10	0.20			0.13	0.50			0.53	1.00	
Krematorij	Positive samples	16			17		3		23	2	3		20	6	8
	Oviposition index	1			1		0.18		0.96	0.08	0.13		0.87	0.26	0.35
	SAI	NA													
	*Ae. albopictus*						1			1	0.67			0.67	0.88
	*Ae. japonicus*								0.09		0.00		0.20		0.25
	*Ae. geniculatus*				0.18				0.09	0			0.35	0.33	
Gračani	Positive samples	20	5		26	3			24	3	1		22	4	2
	Oviposition index	0.87	0.22		1	0.12			0.96	0.12	0.04		1	0.18	0.09
	SAI														
	*Ae. albopictus*		0.40			1				0.67	1			1	1
	*Ae. japonicus*	0.10			0.12				0.08		0		0.18		1
	*Ae. geniculatus*								0.04	0			0.09	0.50	
Markuševec	Positive samples	10	1		18	9			17	3			18	1	1
	Oviposition index	0.91	0.09		0.86	0.43			0.85	0.15			1	0.06	0.06
	SAI														
	*Ae. albopictus*		0			0.67				0.33				1	1
	*Ae. japonicus*	0			0.33				0.06				0.06		0
	*Ae. geniculatus*												0.06	0	
Miroševac	Positive samples	23	1		29	2			27	1			23		1
	Oviposition index	0.96	0.04		1	0.07			0.96	0.04			1		0.04
	SAI														
	*Ae. albopictus*		0			1				1					1
	*Ae. japonicus*	0			0.07				0.04						
	*Ae. geniculatus*												0.04		
Markovo polje	Positive samples	14	8		24	11	1		15	2			12	8	3
	Oviposition index	0.88	0.50		0.96	0.44	0.04		0.94	0.13			0.75	0.50	0.19
	SAI														
	*Ae. albopictus*		0.38			0.91	1			0.50				0.50	1
	*Ae. japonicus*	0.21			0.42		0		0.07				0.33		0.67
	*Ae. geniculatus*				0.04	0							0.25	0.25	
Lučko	Positive samples	17			20				20				25		
	Oviposition index	1			1				1				1		
	SAI	NA			NA				NA				NA		
Brezovica	Positive samples	20			14				17				21		1
	Oviposition index	1			1				1				1		0.05
	SAI	NA			NA				NA						
	*Ae. albopictus*														1
	*Ae. japonicus*														
	*Ae. geniculatus*												0.05		
Stenjevec	Positive samples	24			33	7			24	1			24	1	
	Oviposition index	1			0.97	0.21			1	0.04			1	0.04	
	SAI:	NA													
	*Ae. albopictus*					0.86				1				1	
	*Ae. japonicus*				0.18				0.04				0.04		
	*Ae. geniculatus*														
Gornje Vrapče	Positive samples	22	3		29	2			17	2			18	1	
	Oviposition index	0.96	0.13		1	0.07			0.89	0.11			0.95	0.05	
	SAI:														
	*Ae. albopictus*		0.67			1				0				0	
	*Ae. japonicus*	0.09			0.07				0				0		
	*Ae. geniculatus*														
Šestine	Positive samples	16	1		20	7			13	3			24	1	2
	Oviposition index	0.94	0.06		0.95	0.33			0.81	0.19			1	0.04	0.08
	SAI:														
	*Ae. albopictus*		0			0.86				0				1	1
	*Ae. japonicus*	0			0.30				0				0.04		0.50
	*Ae. geniculatus*												0.08	1	
Sveta Klara	Positive samples	20			19				17				18		
	Oviposition index	1			1				1				1		
	SAI:	NA			NA				NA				NA		

SAI: Species Association Index, NA: not applicable because only *Ae. albopictus* was present. Oviposition index = number of positive samples of one species/total positive samples. Species Association Index = number of samples of observed species in one cemetery collected in the same ovitrap with another species/total number of positive samples of the observed species in the same cemetery.

## Data Availability

No new data were created or analyzed in this study. Data sharing is not applicable to this article.

## Supplementary Materials

The following supporting information can be downloaded at: https://www.mdpi.com/article/10.3390/tropicalmed9110263/s1, Figure S1: The mean daily air temperature in a four-year period (2017-2020) of invasive mosquitoes monitoring in Zagreb; Figure S2: The total amount of rainfall in a four-year period (2017–2020) of invasive mosquitoes monitoring in Zagreb; Figure S3: The mean daily relative humidity in a four-year period (2017–2020) of invasive mosquitoes monitoring in Zagreb; Figure S4: The seasonal dynamics of *Aedes* invasive species during a four-year period (2017–2020) in cemeteries of Zagreb, demonstrated via total (absolute) egg number, POI (%) and MET; Figure S5: Co-habitation of *Ae. albopictus*, *Ae. japonicus* and *Ae geniculatus* in twelve cemetaries in Zagreb, presented in colors for better visual perception of thee species association during four years (2017–2020).
