# Supplementary material for: Temporal Activity and Distribution of the Invasive Mosquitoes Aedes albopictus and Aedes japonicus in the Zagreb Area, Croatia
Source: Trop Med Infect Dis. 2024 Nov 4;9(11):263. doi: 10.3390/tropicalmed9110263 (PMC11598040; doi:10.3390/tropicalmed9110263)
Supplement: Supplementary file 1 [file tropicalmed-09-00263-s001.zip › tropicalmed-3210851-supplementary.pdf]

Tropical Medicine and Infectious Disease

MDPI

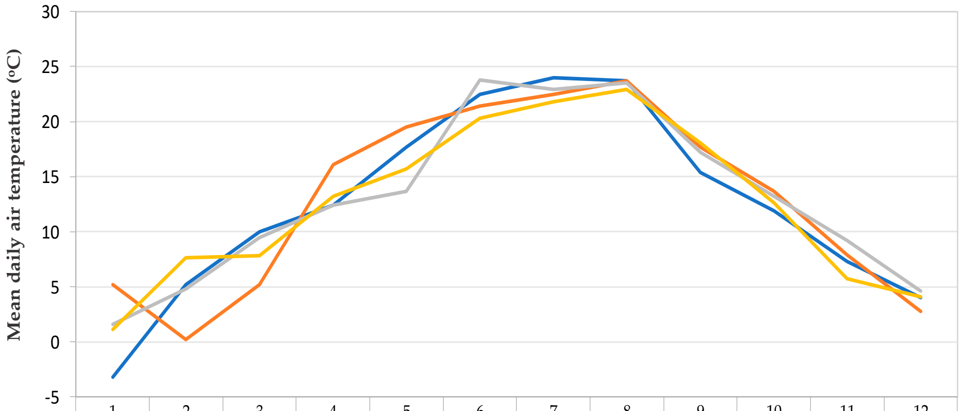

	1	2	3	4	5	6	7	8	9	10	11	12
2017	-3,2	5,2	10	12,4	17,7	22,5	24	23,7	15,4	11,9	7,3	4
2018	5,2	0,2	5,2	16,1	19,5	21,4	22,5	23,7	17,7	13,7	7,9	2,8
2019	1,6	4,8	9,5	12,4	13,7	23,8	22,9	23,5	17,2	13,2	9,2	4,6
2020	1,1	7,6	7,8	13,2	15,7	20,3	21,8	22,9	18,1	12,6	5,7	4,1

Months

Figure S1. The mean daily air temperature in a four-year period (2017-2020) of invasive mosquitoes monitoring in Zagreb.

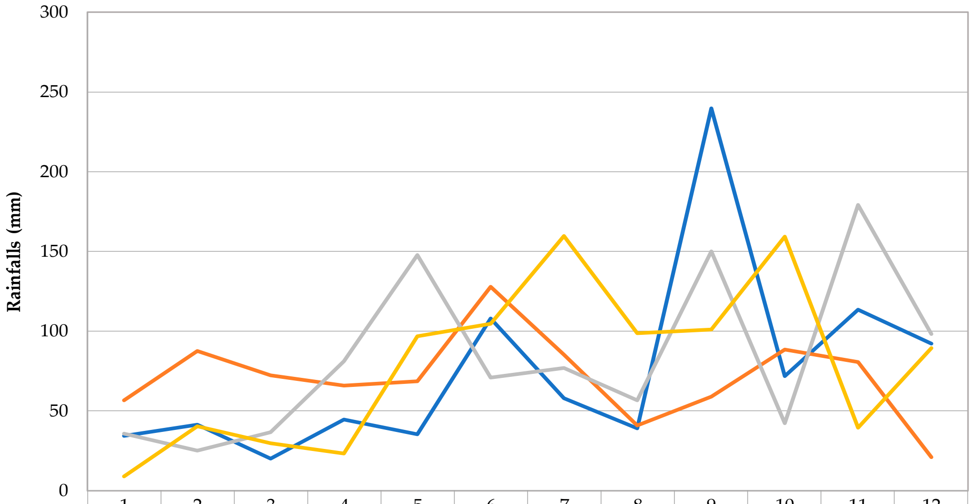

	1	2	3	4	5	6	7	8	9	10	11	12
2017	34,3	41,4	19,8	44,3	35,2	107,8	58	39,1	239,6	72	113,2	92,3
2018	56,7	87,5	72,2	65,8	68,7	127,8	85,2	40,7	59,0	88,6	80,4	21,0
2019	35,8	25,1	36,8	81,1	147,7	70,8	76,8	56,7	150,1	42,3	179,2	98,1
2020	8,7	40,5	29,5	23,4	96,7	104,4	159,6	98,8	100,7	159,3	39,5	89,3

Months

Figure S2. The total amount of rainfall in a four-year period (2017-2020) of invasive mosquitoes monitoring in Zagreb.

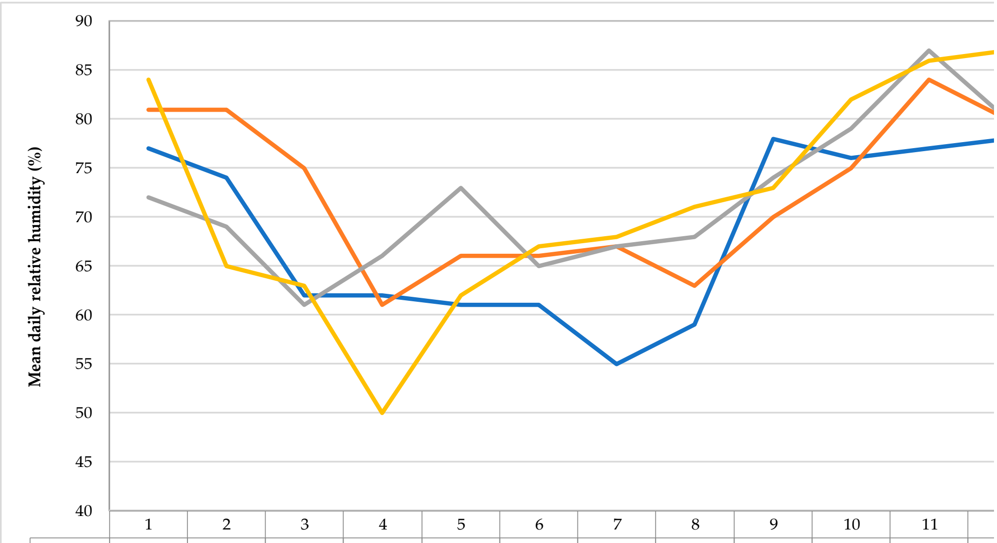

	1	2	3	4	5	6	7	8	9	10	11
2017	77	74	62	62	61	61	55	59	78	76	77
2018	81	81	75	61	66	66	67	63	70	75	84
2019	72	69	61	66	73	65	67	68	74	79	87
2020	84	65	63	50	62	67	68	71	73	82	86

Figure S3. The mean daily relative humidity in a four-year period (2017-2020) of invasive mosquitoes monitoring in Zagreb.

The lowest value   The highest value

	Year	Weeks 17-18	19-20	21-22	23-24	25-26	27-28	29-30	31-32	33-34	35-36	37-38	39-40	41-42	43-44	45-46
Total eggs	2017	0	68	904	1655	2464	4388	4518	3735	4055	2406	506	1064	351	146	13
	2018	88	323	1292	2111	1951	3035	6463	11761	9568	3974	8484	1880	783	205	77
	2019	2	1	68	931	1784	3190	2399	2043	4269	7469	3409	961	578	783	2
	2020	0	71	245	378	577	2612	1706	4896	3899	4659	7056	2852	599	123	0
POI (%)	2017	0.00	8.33	25.00	41.67	55.56	88.89	83.33	88.89	97.22	91.67	47.22	55.56	44.44	36.11	11.43
	2018	5.56	20.59	55.56	68.57	69.44	86.11	97.22	97.22	94.44	86.11	100.00	69.44	50.00	27.78	11.11
	2019	2.78	2.94	8.33	54.55	63.64	88.57	84.85	94.44	100.00	100.00	97.22	76.47	61.76	35.29	2.86
	2020	0.00	5.88	23.53	29.41	57.14	88.57	83.33	97.22	100.00	94.44	97.22	90.91	65.63	18.18	0.00
MET	2017	0.00	22.67	100.44	110.33	123.20	137.13	150.60	116.72	115.86	72.91	29.76	53.20	21.94	11.23	3.25
	2018	44.00	46.14	64.60	87.96	78.04	97.90	184.66	336.03	281.41	128.19	235.67	75.20	43.50	20.50	19.25
	2019	2.00	1.00	22.67	51.72	84.95	102.90	85.68	60.09	118.58	207.47	97.40	36.96	27.52	65.25	2.00
	2020	0.00	35.50	30.63	37.80	28.85	84.26	56.87	139.89	108.31	137.03	201.60	95.07	28.52	20.50	0.00

Figure S4. The seasonal dynamics of *Aedes* invasive species during a four-year period (2017-2020) in cemeteries of Zagreb, demonstrated via total (absolute) egg number, POI (%) and MET.

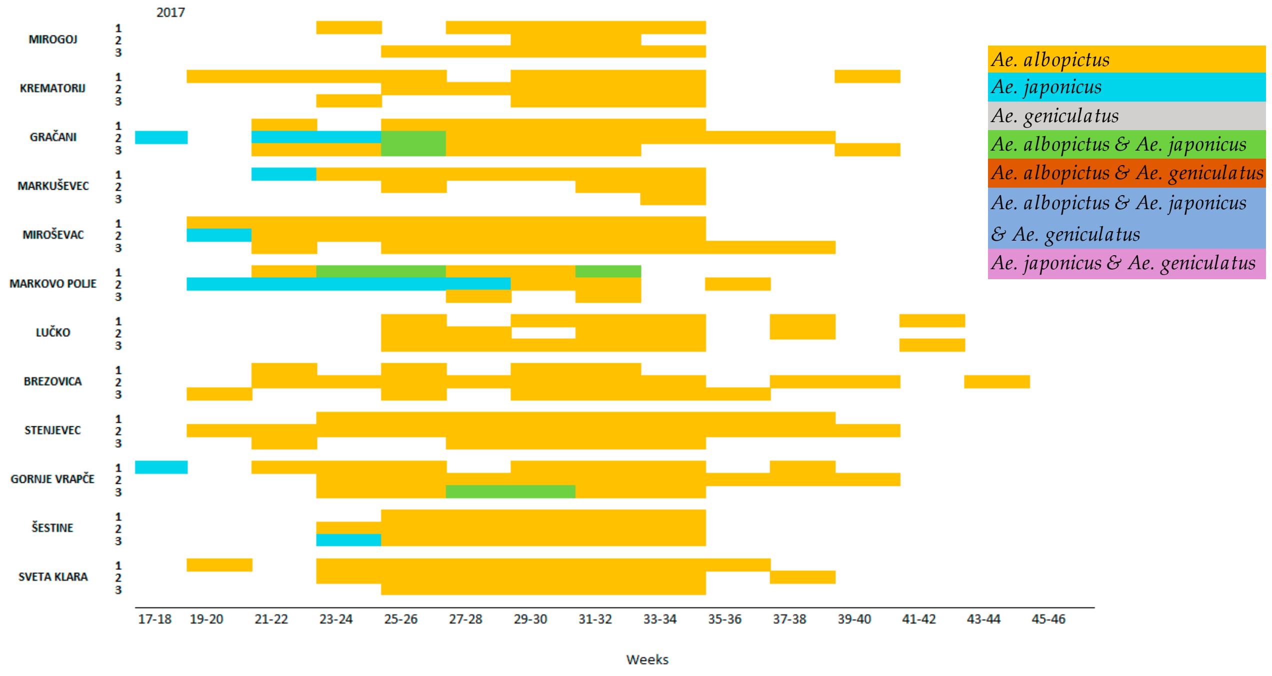
2017
MIROGOJ
KREMATORIJ
GRAČANI
MARKUŠEVEC
MIROŠEVAC
MARKOVO POLJE
LUČKO
BREZOVICA
STENJEVEC
GORNJE VRAPČE
ŠESTINE
SVETA KLARA
1
2
3
17-18
19-20
21-22
23-24
25-26
27-28
29-30
31-32
33-34
35-36
37-38
39-40
41-42
43-44
45-46
Weeks
Ae. albopictus
Ae. japonicus
Ae. geniculatus
Ae. albopictus & Ae. japonicus
Ae. albopictus & Ae. geniculatus
Ae. albopictus & Ae. japonicus & Ae. geniculatus
Ae. japonicus & Ae. geniculatus

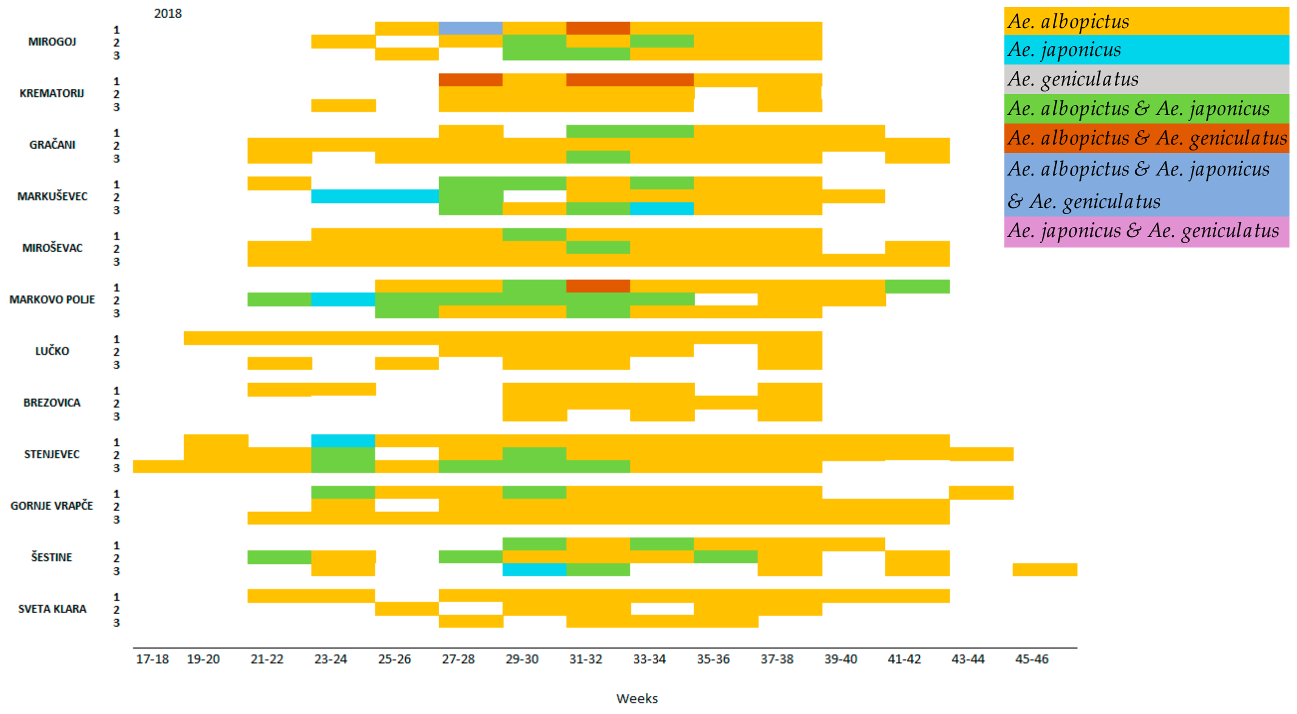
2018
MIROGOJ
KREMATORIJ
GRAČANI
MARKUŠEVEC
MIROŠEVAC
MARKOVO POLJE
LUČKO
BREZOVICA
STENJEVEC
GORNJE VRAPČE
ŠESTINE
SVETA KLARA
1
2
3
17-18
19-20
21-22
23-24
25-26
27-28
29-30
31-32
33-34
35-36
37-38
39-40
41-42
43-44
45-46
Weeks
Ae. albopictus
Ae. japonicus
Ae. geniculatus
Ae. albopictus & Ae. japonicus
Ae. albopictus & Ae. geniculatus
Ae. albopictus & Ae. japonicus & Ae. geniculatus
Ae. japonicus & Ae. geniculatus

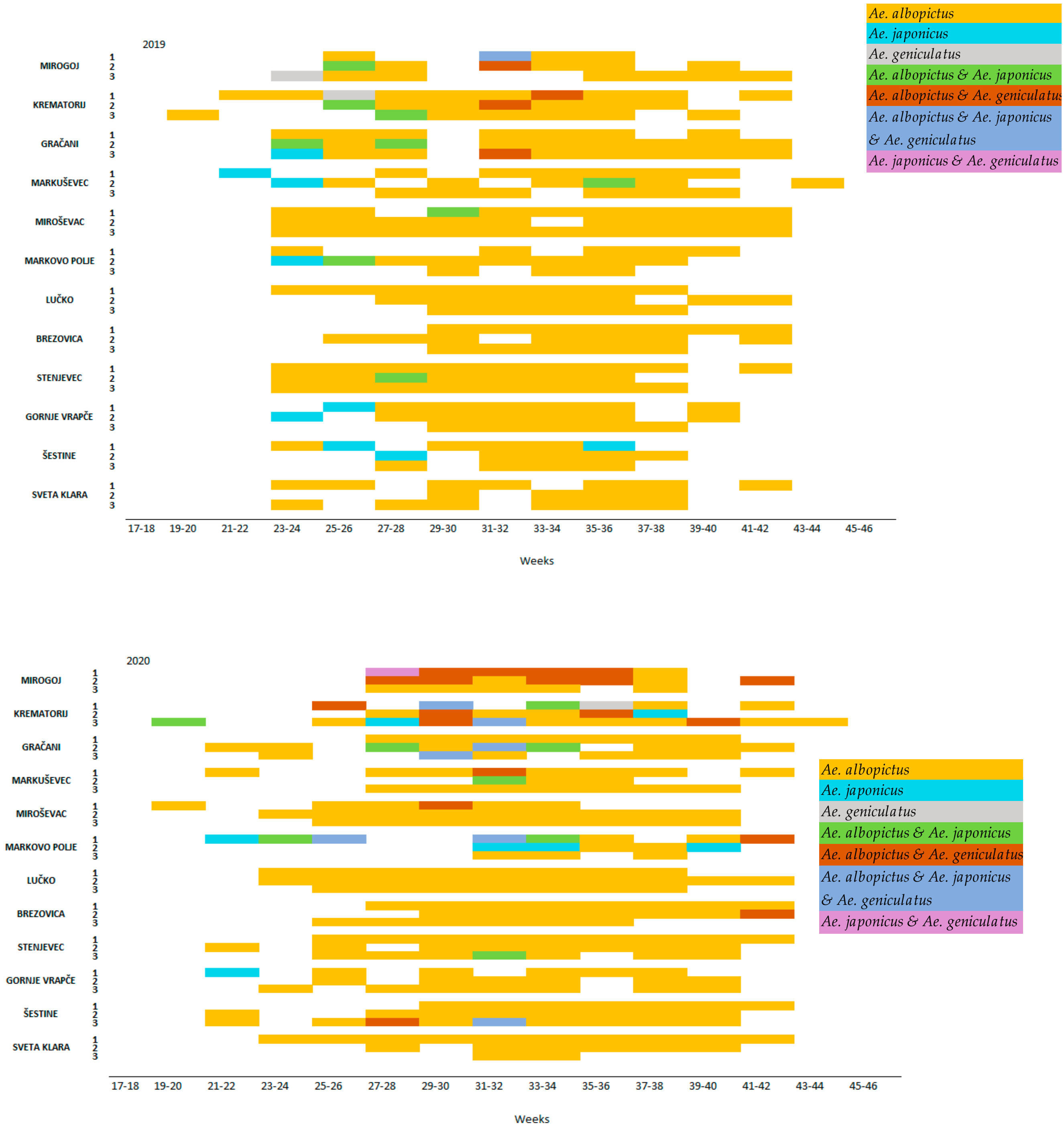

Figure S5. Co-habitation of *Ae. albopictus, Ae. japonicus* and *Ae geniculatus* in twelve cemetaries in Zagreb, presented in colors for better visual perception of thee species association during four years (2017-2020).
